# Pertussis epidemiology in Canada, 2005–2019

**DOI:** 10.14745/ccdr.v49i01a05

**Published:** 2023-01-05

**Authors:** Disha Bhagat, Myriam Saboui, Grace Huang, Francesca Reyes Domingo, Susan G Squires, Marina I Salvadori, Y Anita Li

**Affiliations:** 1 Infectious Disease Program Branch, Public Health Agency of Canada, Ottawa, ON; 2 Health Promotion and Chronic Disease Prevention Branch, Public Health Agency of Canada, Ottawa, ON; 3 Vaccine Rollout TaskForce, Public Health Agency of Canada, Ottawa, ON

**Keywords:** pertussis, whooping cough, Canada, epidemiology, surveillance, vaccination

## Abstract

**Background:**

Pertussis, also known as whooping cough, is an endemic vaccine-preventable disease that affects the respiratory tract and is caused by the bacterium *Bordetella pertussis*. Between 1999 and 2004, the adolescent booster dose of pertussis was introduced across Canada. This report describes the epidemiology of pertussis in Canada from 2005 to 2019, the period after adolescent acellular vaccination was recommended.

**Methods:**

We analyzed pertussis incidence by year, age groups, sex and geographic region using national surveillance data from the Canadian Notifiable Disease Surveillance System. Hospitalization data from the Discharge Abstract Database was used to investigate pertussis hospitalizations by sex and age. Deaths from pertussis were explored using Statistics Canada’s vital statistics data. Vaccination coverage data was gathered from the 2019 Childhood National Immunization Coverage Survey and 2018–2019 Seasonal Influenza Vaccination Coverage Survey.

**Results:**

Between 2005 and 2019, there were a total of 33,481 pertussis cases with the average annual incidence rate of 6.4 cases per 100,000 population. The highest average age-specific incidence rate was among infants under one year of age (n=68.7 cases per 100,000 population). There were a total of 1,593 pertussis hospitalizations; nearly 80% of these hospitalizations were infants under one year of age. Hospitalization rates were 8.2 times higher in infants three months or younger compared to infants four to 11 months of age. There were 17 deaths; all among infants under one year of age.

**Conclusion:**

The highest morbidity and fatality of pertussis were among infants under one year of age. It is important to take measures to reduce transmission to infants who are too young to be vaccinated. Increasing vaccine coverage in children and pregnant women are important to reduce the burden of disease.

## Introduction

Pertussis, also known as whooping cough, is an infectious disease affecting the respiratory tract and is caused by the bacterium *Bordetella pertussis* (1)). Although pertussis is a vaccine-preventable disease, it is endemic worldwide, including in Canada. Pertussis has been under national surveillance in Canada since 1924. Since 1943, national childhood vaccination programs have been available and have contributed to a significant reduction in the incidence of pertussis ((1)). Currently, there are publicly funded routine vaccination programs for infants, adolescents, pregnant women and adults across Canada; however, there are differences in vaccine products administered and the recommended vaccination schedules between some provinces and territories ((2,3)). Between 1999 and 2004, the adolescent booster dose of pertussis, at 14 to 16 years of age, was introduced across Canada ((4)). In 2018, the National Advisory Committee for Immunization recommended a dose of the tetanus, diphtheria and pertussis (Tdap) vaccination be offered with every pregnancy, as immunization during pregnancy is a method to provide passive protection through antibody transfer to the infant ((5)). The purpose of this report is to provide a summary of the epidemiology of pertussis in Canada between 2005 and 2019; the period following the implementation of the adolescent acellular pertussis vaccination programs.

## Methods

### National case reports

Nationally reported confirmed cases of pertussis from 2005 to 2019 were extracted from the Canadian Notifiable Diseases Surveillance System (CNDSS) database in June 2021 ((6)). National pertussis case definitions were updated in 2009 ((7)). Between 2000 and 2008, a confirmed pertussis case was defined as laboratory confirmation of infection or an epidemiological link to a laboratory-confirmed cases and the presentation of at least one of a list of clinical symptoms ((8)). In 2009, the case definition was further refined to require those cases in which *B. pertussis* deoxyribonucleic acid was detected to also have clinically compatible symptoms ((7)).

### Hospitalizations

Hospitalization data from 2005 to 2019 were obtained from the Canadian Institute for Health Information’s Discharge Abstract Database (DAD) and were extracted in November 2021 ((9)). The International Classification of Diseases, Tenth Modification (ICD-10) was used for coding diagnoses. Records in DAD with the most responsible diagnosis code of either A37.0 (Whooping cough due to *Bordetella pertussis*) or A37.9 (Whooping cough, unspecified) were included. Hospital transfers and readmissions that occurred within six weeks of admission were excluded. The DAD includes pertussis hospitalizations of all acute hospital discharges in Canada, with the exception of Québec ((9)).

### Deaths

Mortality data were obtained from Statistics Canada’s Death Database; a national mortality database collected annually ((10)). Deaths with an underlying cause of pertussis were identified using the same ICD-10 codes listed above.

### Vaccinations

The 2019 Childhood National Immunization Coverage Survey was used to obtain national childhood vaccination coverage data. This survey is conducted on a biennial basis by Statistics Canada on behalf of the Public Health Agency of Canada to estimate national uptake for all publicly funded routine childhood vaccinations, including tetanus, diphtheria and acellular pertussis vaccines ((11)). The 2019 survey included the Survey on Vaccination during Pregnancy, in which the biological mothers of children born from September 1, 2018, to March 1, 2019, were surveyed regarding pertussis vaccination during their pregnancy ((12)). National adult vaccination coverage estimates were from the 2018–2019 Seasonal Influenza Vaccination Coverage Survey, which includes adult vaccination coverage for the TdaP vaccine booster ((13)).

### Analysis

Incidence rates and hospitalization rates of pertussis were calculated per 100,000 population using Statistics Canada population data ((14)). To explore geographic distributions, Canada was divided into four main regions—Northern, Atlantic, Western and Central. The Northern region included Yukon, Northwest Territories and Nunavut. The Atlantic region included New Brunswick, Nova Scotia, Prince Edward Island and Newfoundland and Labrador. The Western region included British Columbia, Alberta, Saskatchewan and Manitoba. Lastly, the Central region included Ontario and Québec. Age-standardized incidence rates were used to compare geographic regions. These rates were calculated using the direct method with the 2011 Canadian population as the standard. Cases with missing age were excluded from the age standardization. Fewer than 2% of cases had missing ages.

As hospitalization numbers did not include the province of Québec, the proportion of cases hospitalized and hospitalization rates were calculated excluding the cases and population of Québec, respectively. For hospitalization rates of infants under one year of age, age groups were created in accordance with National Advisory Committee for Immunization’s recommendations to administer a pertussis vaccine at two, four, six and 12–23 months ((15)). Thus, the age groupings for infants under one year were as follows: under two months, two to three months, four to five months and six to 11 months. Population estimates for one-month interval ages for infants under one year of age were not available to calculate hospitalization rates; therefore, the denominator for each one-month age interval was estimated by dividing the population under one year of age by 12, resulting in each infant contributing one month to the rate.

Negative binomial regression was used to estimate the association between rates, sex, age groups, and time periods. Statistical significance was considered at a confidence level of 95%. All statistical analyses were conducted using SAS 9.4.

## Results

### Cases and incidence

**Figure 1:**
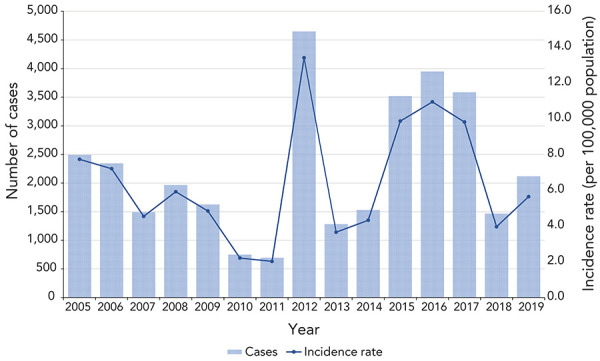
Reported cases and incidence rate, per 100,000 population, of pertussis in Canada by year, 2005–2019

From 2005 to 2019, a total of 33,481 cases of pertussis were reported, with an average annual incidence rate per 100,000 population of 6.4 (range: 2.0 in 2011 to 13.4 in 2012) (**Figure 1**). Pertussis incidence peaked in the years 2012 and 2015 to 2017. During this 15-year period, females accounted for an average of 54.2% of cases while males accounted for 45.7% of cases. The difference in incidence rates between sex was not statistically significant (*p*-value=0.40).

**Figure 2:**
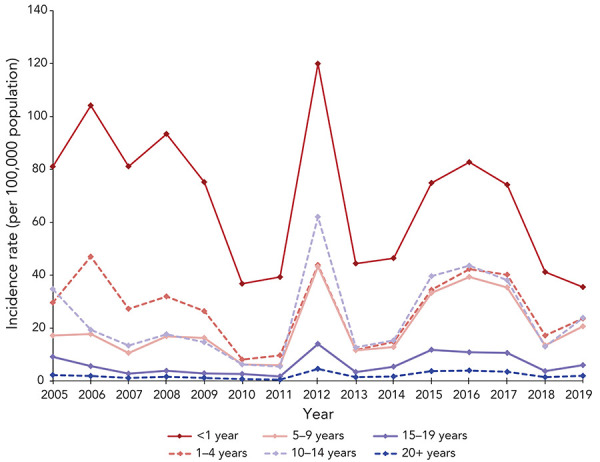
Incidence rate, per 100,000 population, of pertussis reports in Canada by age group^a^ by year, 2005–2019 ^a^ Age group in years

Infants under one year of age had the highest average incidence rate per 100,000 population by age group (n=68.7) and accounted for 13.1% of cases. Following the under one year age group, the highest average annual incidence rates were among the one to four years of age (n=27.2), 10 to 14 years of age (n=24.0), and five to nine years of age (n=20.1). The average incidence rate was lower in the 15 to 19-year age group (n=6.3) and the lowest average rate was among adults over 20 years of age (n=2.1). The trends in pertussis incidence were similar in age groups covering children under 15 years of age, with peaks in 2012 and 2015–2017 (**Figure 2**). There were also peaks in incidence that were specific to age groups such as a peak in the years 2005 to 2009 for the under one-year age group and a peak in 2006 for the one to four-year age group.

**Figure 3:**
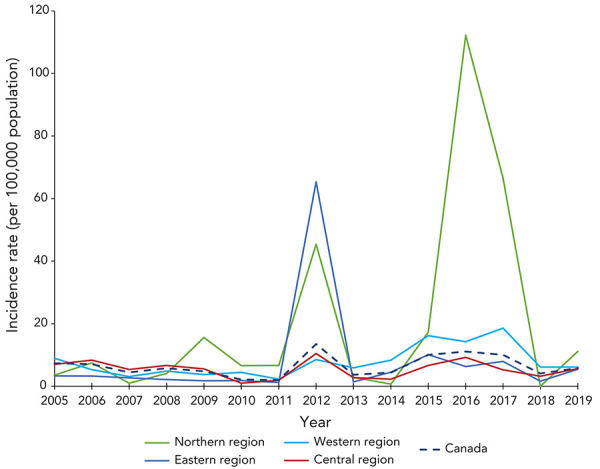
Trends in annual age-standardized incidence rate of pertussis, per 100,000 population, by geographic region and nationwide, 2005–2019

Variations in pertussis age-standardized incidence among the four geographic regions of Canada were observed (**Figure 3**). The average annual age-standardized incidence of each region is not statistically different from the other regions nor from the national incidence. The age-standardized incidence rate per 100,000 population of pertussis fluctuated less in the Central region, where the average was 5.4 (95% CI: 3.9–7.0) and the Western region, where the average was 7.8 (95% CI: 5.1–10.5). The greatest fluctuations in pertussis incidence and the highest peaks occurred in the Northern region, where the average was 20.1 (95% CI: 2.7–37.5). The Atlantic region observed fairly steady incidence rates with an average incidence rate of 7.9 (95% CI: -1.0–16.9), with the exception of 2012. In 2012, the Atlantic region observed a 55-fold increase in the incidence rate compared to the previous year when the region accounted for 31% of the nation’s cases.

### Hospitalizations

**Figure 4:**
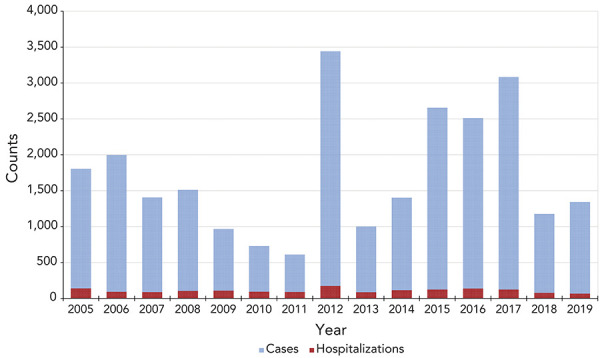
Number of pertussis cases and hospitalizations^a^ in Canada by year, 2005–2019 ^a^ Cases and hospitalizations exclude the province of Québec

From 2005 to 2019, there were a total of 1,593 acute care pertussis hospitalizations, averaging 106.2 hospitalizations each year (range: 66 in 2019 to 173 in 2012) (**Figure 4**). The average hospitalization rate per 100,000 population was 0.4 per year (range: 0.2 in 2019 to 0.6 in 2012). Although case data from CNDSS and hospitalization data from DAD cannot be linked, the proportion of pertussis cases that were hospitalized was estimated as less than 10% for this 15-year period. The trends in hospitalizations followed the trends in pertussis cases as the peaks in hospitalization counts occurring in 2012 and 2015 to 2017 coincided with the peaks in cases. During this period, females accounted for an average 57.7% of hospitalizations while males accounted for 48.5%; a difference that was not statistically significant (*p*-value=0.96).

**Figure 5:**
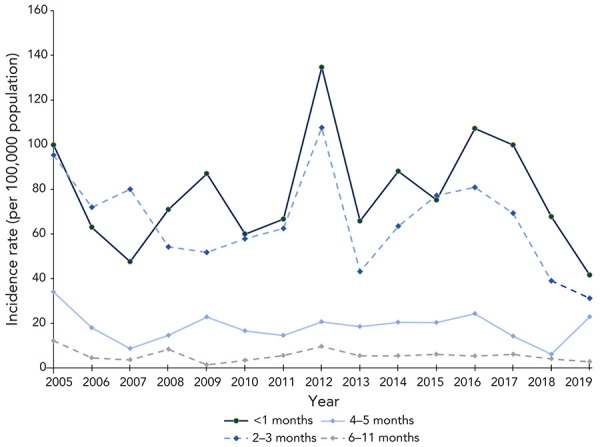
Hospitalization rate of infants under one year of age, by age group, 2005–2019

Infants under one year of age accounted for over 80% of all hospitalizations and had an average hospitalization rate per 100,000 population of 29.9 (range: 17.3 in 2019 to 48.6 in 2012). Focusing specifically on the under one year of age population, the hospitalization rate was 8.2 times higher (95% CI: 6.4–10.4; *p*-value <0.01) in infants under four months of age compared to infants four to 11 months of age over the 15-year period (**Figure 5**).

In 2019, following the 2018 recommendation for pertussis vaccination during pregnancy, there was a decline in hospitalization rates among younger infants, compared to the period of 2005 to 2018; the years before the recommendation. There was a two-fold decline in the hospitalization rate in 2019 compared to the mean hospitalization rate in the period of 2005 to 2018 among infants under two months of age (95% CI: 1.1–3.4; *p*-value=0.02) and among infants between two and three months of age (95% CI: 1.3–4.0; *p*-value=0.01). In contrast, the decline was not significant among infants between four and five months of age (*p*-value=0.51) and six to 11 months of age (*p*-value=0.24).

### Deaths

From 2005 to 2019, Statistics Canada reported 17 deaths with pertussis listed as the leading cause of death, with zero to three deaths reported each year. All 17 deaths were infants under one year of age. Nine deaths occurred in females (52.9%) and eight deaths in males (47.1%); a difference that was not significant (*p*-value=0.81).

### Vaccination

The 2019 Canadian Childhood National Immunization Coverage Survey estimated the vaccine coverage rate for at least four doses of the tetanus, diphtheria, and pertussis vaccine administered by two years of age was 78%, for at least five doses by seven years was 78%, and for one booster dose by 17 years was 95% ((11)). This survey also revealed that, of the mothers who knew whether or not they had been vaccinated against pertussis during their pregnancy, 44% had been vaccinated ((12)). The coverage among adults was lower, with only 33% of adults over 18 years of age having received a pertussis booster in adulthood, as estimated by the 2018–2019 Seasonal Influenza Vaccination Coverage Survey ((13)).

## Discussion

Since the introduction of the pertussis vaccine and routine pertussis vaccination programs in Canada in 1943, the national incidence rate of pertussis decreased overall ((16)). Our study shows that since 2004, when the vaccination recommendation of at least one adult dose was introduced, there was not a steady increase or decrease in the pertussis incidence, but rather fluctuations with peaks on a national and regional level. Importantly, the greatest burden of pertussis was observed among the under one year of age population, which consistently had the highest annual incidence rate, accounting for nearly 80% of hospitalizations, and all 17 pertussis-related deaths during this 15-year period. In contrast, adults over 20 years of age had the lowest annual incidence rate each year.

Sporadic peaks in pertussis incidence were observed in each geographic region. Although data available on pertussis cases does not include outbreak information, peaks in incidence in a geographic region can be connected to outbreaks occurring at a regional level. For example, in 2012, a 55-fold increase in the incidence rate in the Atlantic region was associated with a large outbreak declared in New Brunswick, involving 1,421 confirmed cases, in which 2% of the cases were hospitalized. Over half of the cases were among school-aged children, with the 10 to 14-year-old age group followed by the five to nine-year-old age group having the highest age-specific incidence rates. Province-wide school-based immunization campaigns were implemented in spring and fall of 2012 to battle this large outbreak ((17)). From 2005 to 2019, the highest regional peaks in incidence occurred in the Northern region, which can be attributed to large outbreaks that were further accentuated by the smaller population in the territories compared with the other geographic regions. The spike in incidence, in the Northern region in 2016 and 2017, was connected to a large outbreak in Nunavut occurring in May 2016 to April 2017, which spread to 11 communities with 163 confirmed cases and a second smaller outbreak occurring in September to November of 2017 ((6,18)). Sporadic peak years in pertussis activity have been reported in other countries including the United States, Greece, Finland and Singapore, to name a few, in the past several decades ((19,20)). Studies have hypothesized that the cyclic nature of this disease could be attributable to low vaccination numbers, waning vaccine immunity and changes in *Bordetella pertussis* ((21–23)).

Hospitalization data obtained from DAD does not include the province of Québec. According to the *Maintenance et exploitation des données pour l’étude de la clientèle hospitalière* (Med-Echo), a clinical administrative Québec database, a total of 710 pertussis hospitalizations were recorded in Québec between April 1, 2006, and March 31, 2020 ((24)). During the same period, 1,448 hospitalizations were recorded in DAD. While the population of Québec accounts for about 23% of Canada’s population, approximately 33% of pertussis hospitalizations in Canada occurred in Québec during this study period and were not captured in this study.

The greatest burden of disease was among infants under one year of age. Hospitalization rates were over eight times higher in infants under four months of age than infants between four and 11 months, which coincides with the first dose of the pertussis vaccine administered at two months of age. Three different studies, including a Canada-wide study by Desai *et al.* ((25)), a study covering British Columbia and Québec by Skowronski *et al.* ((26)) and a study in the United States by Masseria *et al.* ((27)), all reported similar findings showing pertussis hospitalizations were highest in infants under three months of age. The lower hospitalization rates among infants between four and 11 months of age, compared to infants under four months of age, can be attributed to less severe disease in older infants and to vaccinations. This highlights the importance of timely childhood immunization.

The 2019 national vaccination coverage estimates show that childhood vaccine uptake could be improved, as both childhood vaccine coverage goals of 95% coverage of four or more doses by two years of age and 95% coverage of five or more doses by seven years of age have not been reached ((28)). In contrast, the national coverage goal of 90% coverage for the adolescent booster by 17 years of age was met ((11,28)).

Adult vaccine coverage for pertussis in 2019 was also low, with only 33% of all adults having received a pertussis booster, which was the lowest among all vaccines covered by publicly funded programs, despite national recommendations to receive a booster dose of a pertussis-containing vaccine ((13)). Findings from a 2015 study surveying over 1,000 healthcare providers across Canada indicated low levels of knowledge among healthcare providers about Tdap recommendations in adults, resulting in a low likelihood of providing Tdap recommendations to patients in accordance with national recommendations ((29)). Although the incidence rate per 100,000 of pertussis among adults was low at 2.1, pertussis is a highly communicable disease resulting in a significant risk of transmission from infected adults to infants under one year of age who are at highest risk of pertussis-related complications, hospitalizations and death ((1)). This indicates a need to improve awareness of adult vaccinations among healthcare providers and the public to improve adult vaccination uptake. It is anticipated that higher vaccine coverage among children and adults could decrease pertussis activity within the vaccinated population and also decrease transmission of infection to young infants who are unimmunized and have the greatest morbidity and mortality from the disease ((30)).

In February 2018, Tdap vaccination was recommended in every pregnancy and by November 2019, all provinces and territories except for Ontario and British Columbia implemented publicly funded pertussis vaccination with each pregnancy ((5,31)). Within a year following this recommendation, vaccine coverage among pregnant women was low at 44%, but coverage rates are expected to increase with time as more provinces and territories fund maternal vaccination and the recommendation becomes more well known ((12,31)). As of April 2022, all provinces and territories have publicly funded Tdap vaccination during each pregnancy ((2,32)). Although more time is needed to evaluate the potential benefits of maternal pertussis vaccination during pregnancy in Canada, our analysis shows early signs of a significant decrease in pertussis hospitalization rates among infants under four months of age in 2019 compared to the mean hospitalization rates in 2005 to 2018. In addition, in 2019, there was a decrease in pertussis hospitalization rates among infants under four months of age, despite a national increase in hospitalization rates for all ages, compared to the previous year. Further insight can be gained from other countries with maternal vaccination. A 2020 study in Brazil by Friedrich *et al.* found a 47.7% decrease in the mean annual incidence of pertussis in children under one month of age in the period after maternal pertussis vaccination was implemented compared to the period before maternal vaccination ((33)). As the greatest burden of pertussis is among young infants who are too young to be vaccinated, it is important to couple vaccination coverage numbers with strong surveillance data to assess the impact of recommendations for immunization during pregnancy.

## Limitations

There are several limitations to these findings as data collected from these surveillance systems have constraints. Due to the passive nature of CNDSS, there is a likelihood of underreporting of cases ((6)). A diagnosis of pertussis requires a high level of clinical suspicion, resulting in many cases in both children and adults being undiagnosed. It is likely that the incidence of pertussis is higher among adults than reported, as symptoms are generally milder, and testing for *B. pertussis* among adults is infrequent. Furthermore, trends of incidence rates must be interpreted with caution because of changes in case definition, provincial and territorial reporting and laboratory technologies.

In addition, the pertussis-related hospitalizations are coded based on the physician’s diagnosis and do not necessarily match the national case definition. As a result, the number of pertussis-related hospitalizations from DAD featured in this report may be an underestimate of the actual burden. Furthermore, DAD does not include the province of Québec, which accounts for an estimated one third of pertussis hospitalizations in Canada. However, we speculate that the pertussis hospitalizations in Québec would not change the overall interpretations.

The 2016 to 2019 data on deaths obtained from Statistics Canada’s Death Database were considered preliminary because there were improvements in methodology and timeliness resulting in a shortened data collection period compared with previous years. Thus, there may be fewer deaths captured. In addition, death data for Yukon from 2017 to 2019 were not available ((10)).

Vaccination coverage surveys undergo methodology changes, so coverage estimates cannot be compared between iterations. Furthermore, Childhood National Immunization Coverage Survey data are collected from parent-held vaccination records in which some information may be incomplete, erroneous or missing, leading to an underestimate of vaccination coverage ((34)). Neither the CNDSS nor DAD data includes vaccination history of cases or patients, so analysis of vaccine efficacy was limited.

## Conclusion

Following the period after the implementation of an adolescent pertussis booster, pertussis remained an endemic disease in Canada that affected individuals of all ages. However, the greatest burden continued to be among infants under one year of age, especially infants three months and younger who are too young to be vaccinated or have only received one dose of the vaccine. Thus, it is important to prevent transmission of infection by increasing vaccine coverage to protect those at highest risk of severe outcomes. It will be important to monitor the effect of the maternal pertussis vaccination recommendation on epidemiology of infants under four months of age. Enhanced surveillance systems that capture vaccine history, pertussis strains and outbreak information would provide a more comprehensive understanding of pertussis epidemiology that can be used to assess vaccine recommendation changes and to inform further public health action.

## References

[1] Public Health Agency of Canada. Pertussis (whooping cough): for health professionals. Ottawa, ON: PHAC; 2020. [Accessed 2021 March 20]. https://www.canada.ca/en/public-health/services/immunization/vaccine-preventable-diseases/pertussis-whooping-cough/health-professionals.html

[2] Public Health Agency of Canada. Provincial and territorial routine and catch-up vaccination schedule for infants and children in Canada. Ottawa, ON: PHAC; 2020. [Accessed 2021 March 10]. https://www.canada.ca/en/public-health/services/provincial-territorial-immunization-information/provincial-territorial-routine-vaccination-programs-infants-children.html

[3] Public Health Agency of Canada. Provincial and Territorial Routine Vaccination Programs for Healthy, Previously Immunized Adults. Ottawa, ON: PHAC; 2020. [Accessed 2021 June 23]. https://www.canada.ca/en/public-health/services/provincial-territorial-immunization-information/routine-vaccination-healthy-previously-immunized-adult.html

[4] Halperin SA. Canadian experience with implementation of an acellular pertussis vaccine booster-dose program in adolescents: implications for the United States. Pediatr Infect Dis J 2005;24(6 Suppl):S141–6. 10.1097/01.inf.0000166163.02135.8b 15931142

[5] Public Health Agency of Canada. An Advisory Committee Statement (ACS) National Advisory Committee on Immunization (NACI). Update on Immunization in Pregnancy with Tdap Vaccine. Ottawa, ON: PHAC; [Modified 2019 Oct 9; accessed 2021 July 20]. https://www.canada.ca/en/public-health/services/publications/healthy-living/update-immunization-pregnancy-tdap-vaccine.html

[6] Public Health Agency of Canada. Notifiable diseases online. Ottawa, ON: PHAC; [Modified 2021 July 20; accessed 2021 June 10]. https://diseases.canada.ca/notifiable/

[7] Public Health Agency of Canada. Case definitions for diseases under national surveillance. Can Com Dis Rep 2009;35(S2):65–8. Ottawa, ON: PHAC; [Modified 2011 May 9]. https://www.canada.ca/en/public-health/services/reports-publications/canada-communicable-disease-report-ccdr/monthly-issue/2009-35/definitions-communicable-diseases-national-surveillance.html

[8] Health Canada. Case definitions for diseases under national surveillance. Can Commun Dis Rep 2000;26 Suppl 3:i-iv 1–122. https://publications.gc.ca/collections/collection_2016/aspc-phac/HP3-1-26-S3-eng.pdf 11055080

[9] Canadian Institute for Health Information. Discharge Abstract Database metadata (DAD). [Accessed 2021 July 3]. https://www.cihi.ca/en/discharge-abstract-database-metadata-dad

[10] Statistics Canada. Table 13-10-0141-01. Deaths, by cause, Chapter I: Certain infectious and parasitic diseases (A00 to B99). Ottawa, ON: StatCan; 2021. [Accessed 2021 June 18]. 10.25318/1310014101-eng 10.25318/1310014101-eng

[11] Public Health Agency of Canada. Highlights from the 2019 childhood National Immunization Coverage Survey (cNICS). Ottawa, ON: PHAC; [Modified 2022 Feb 7; accessed 2021 July 5]. https://www.canada.ca/en/public-health/services/publications/vaccines-immunization/2019-highlights-childhood-national-immunization-coverage-survey.html

[12] Public Health Agency of Canada. Results of the Survey on Vaccination during Pregnancy. Ottawa, ON: PHAC; 2021. [Accessed 2021 Oct 29]. https://www.canada.ca/en/public-health/services/publications/vaccines-immunization/survey-vaccination-during-pregnancy.html

[13] Public Health Agency of Canada. Vaccine uptake in Canadian adults 2019. Ottawa, ON: PHAC; [Modified 2022 July 11; accessed 2021 July 5]. https://www.canada.ca/en/public-health/services/publications/healthy-living/2018-2019-influenza-flu-vaccine-coverage-survey-results.html

[14] Statistics Canada. Table 17-10-0005-01 Population estimates on July 1^st^, by age and sex. [Accessed 2021 July 21]. 10.25318/1710000501-eng 10.25318/1710000501-eng

[15] Public Health Agency of Canada. Recommended immunization schedules: Canadian Immunization Guide. Ottawa, ON: PHAC; 2020. [Accessed 2021 May 17]. https://www.canada.ca/en/public-health/services/publications/healthy-living/canadian-immunization-guide-part-1-key-immunization-information/page-13-recommended-immunization-schedules.html

[16] Smith T, Rotondo J, Desai S, Deehan H. Pertussis Surveillance in Canada: trends to 2012. Can Commun Dis Rep 2014;40(3):21–30. 10.14745/ccdr.v40i03a01 29769879 PMC5864426

[17] New Brunswick Department of Health. Pertussis outbreak investigation report. Fredericton, NB: Government of New Brunswick; 2014. https://www2.gnb.ca/content/dam/gnb/Departments/h-s/pdf/en/CDC/HealthProfessionals/PertussisReport.pdf

[18] CBC News. Nunavut's 2^nd^ recent whooping cough outbreak is declared over. Nov 22, 2017. https://www.cbc.ca/news/canada/north/whooping-cough-second-outbreak-over-2017-1.4413857

[19] Choisy M, Rohani P. Changing spatial epidemiology of pertussis in continental USA. Proc Biol Sci 2012;279(1747):4574–81. 10.1098/rspb.2012.1761 23015623 PMC3479730

[20] Tan T, Dalby T, Forsyth K, Halperin SA, Heininger U, Hozbor D, Plotkin S, Ulloa-Gutierrez R, Wirsing von König CH. Pertussis Across the Globe: Recent Epidemiologic Trends From 2000 to 2013. Pediatr Infect Dis J 2015;34(9):e222–32. 10.1097/INF.0000000000000795 26376316

[21] Esposito S, Stefanelli P, Fry NK, Fedele G, He Q, Paterson P, Tan T, Knuf M, Rodrigo C, Weil Olivier C, Flanagan KL, Hung I, Lutsar I, Edwards K, O’Ryan M, Principi N; World Association of Infectious Diseases and Immunological Disorders (WAidid) and the Vaccine Study Group of the European Society of Clinical Microbiology and Infectious Diseases (EVASG). Pertussis Prevention: Reasons for Resurgence, and Differences in the Current Acellular Pertussis Vaccines. Front Immunol 2019;10:1344. 10.3389/fimmu.2019.01344 31333640 PMC6616129

[22] Fisman DN, Tang P, Hauck T, Richardson S, Drews SJ, Low DE, Jamieson F. Pertussis resurgence in Toronto, Canada: a population-based study including test-incidence feedback modeling. BMC Public Health 2011;11:694. 10.1186/1471-2458-11-694 21899765 PMC3189138

[23] Tsang RS, Shuel M, Jamieson FB, Drews S, Hoang L, Horsman G, Lefebvre B, Desai S, St-Laurent M. Pertactin-negative Bordetella pertussis strains in Canada: characterization of a dozen isolates based on a survey of 224 samples collected in different parts of the country over the last 20 years. Int J Infect Dis 2014;28:65–9. 10.1016/j.ijid.2014.08.002 25244999

[24] Ministère de la Santé et des Services sociaux. Sources de données et métadonnées - MedEcho. Québec, QC; MSSS; 2016. [Accessed 2022 Jan 25]. https://www.msss.gouv.qc.ca/professionnels/documentation-sources-de-donnees-et-indicateurs/sources-de-donnees-et-metadonnees/med-echo/

[25] Desai S, Schanzer DL, Silva A, Rotondo J, Squires SG. Trends in Canadian infant pertussis hospitalizations in the pre- and post-acellular vaccine era, 1981-2016. Vaccine 2018;36(49):7568–73. 10.1016/j.vaccine.2018.10.047 30392765

[26] Skowronski DM, Janjua NZ, Tsafack EP, Ouakki M, Hoang L, De Serres G. The number needed to vaccinate to prevent infant pertussis hospitalization and death through parent cocoon immunization. Clin Infect Dis 2012;54(3):318–27. 10.1093/cid/cir836 22156850

[27] Masseria C, Martin CK, Krishnarajah G, Becker LK, Buikema A, Tan TQ. Incidence and Burden of Pertussis Among Infants Less Than 1 Year of Age. Pediatr Infect Dis J 2017;36(3):e54–61. 10.1097/INF.0000000000001440 27902648 PMC5312729

[28] Public Health Agency of Canada (PHAC). Vaccination Coverage Goals and Vaccine Preventable Disease Reduction Targets by 2025. Ottawa, ON: PHAC; 2019. [Accessed 2021 July 16]. https://www.canada.ca/en/public-health/services/immunization-vaccine-priorities/national-immunization-strategy/vaccination-coverage-goals-vaccine-preventable-diseases-reduction-targets-2025.html

[29] MacDougall D, Halperin BA, MacKinnon-Cameron D, Li L, McNeil SA, Langley JM, Halperin SA. Universal tetanus, diphtheria, acellular pertussis (Tdap) vaccination of adults: what Canadian health care providers know and need to know. Hum Vaccin Immunother 2015;11(9):2167–79. 10.1080/21645515.2015.1046662 26090861 PMC4635841

[30] National Advisory Committee on Immunization. Prevention of pertussis in adolescents and adults. Can Commun Dis Rep 2003;29(ACS-5):1–9. https://pubmed.ncbi.nlm.nih.gov/14526692/14526692

[31] Gilbert NL, Guay M, Kokaua J, Lévesque I, Castillo E, Poliquin V. Pertussis vaccination in Canadian pregnant women, 2018-2019. J Obstet Gynaecol Can 2022;44(7):762–8. 10.1016/j.jogc.2022.01.014 35151906

[32] Government of Ontario. Ontario's routine immunization schedule. Toronto, ON: Government of Ontario. [Accessed 2022 April 1]. https://www.health.gov.on.ca/en/public/programs/immunization/static/immunization_tool.html#pregnancy

[33] Friedrich F, Valadão MC, Brum M, Comaru T, Pitrez PM, Jones MH, Pinto LA, Scotta MC. Impact of maternal dTpa vaccination on the incidence of pertussis in young infants. PLoS One 2020;15(1):e0228022. 10.1371/journal.pone.0228022 31990945 PMC6986709

[34] Public Health Agency of Canada. Vaccine coverage in Canadian children: Results from the 2017 Childhood National Immunization Coverage Survey (CNICS). Ottawa, ON: PHAC; [Modified 2020 Jan 29]. https://www.canada.ca/en/public-health/services/publications/healthy-living/2017-vaccine-uptake-canadian-children-survey.html

